# Estimation of effective population size using single-nucleotide polymorphism (SNP) data in Jeju horse

**DOI:** 10.1186/2055-0391-56-28

**Published:** 2014-12-05

**Authors:** Kyoung-Tag Do, Joon-Ho Lee, Hak-Kyo Lee, Jun Kim, Kyung-Do Park

**Affiliations:** Department of Equine Sciences, Sorabol College, Gyeongju, 780-711 Republic of Korea; The Animal Genomics and Breeding Center, Hankyong National University, Anseong, 456-749 Republic of Korea; Provincial Livestock Promotion, Jeju, 690-802 Republic of Korea

**Keywords:** Jeju horse, Linkage disequilibrium (LD), Effective population size

## Abstract

This study was conducted to estimate the effective population size using SNPs data of 240 Jeju horses that had raced at the Jeju racing park. Of the total 61,746 genotyped autosomal SNPs, 17,320 (28.1%) SNPs (missing genotype rate of >10%, minor allele frequency of <0.05 and Hardy–Weinberg equilibrium test P-value of <10^–6^) were excluded after quality control processes. SNPs on the X and Y chromosomes and genotyped individuals with missing genotype rate over 10% were also excluded, and finally, 44,426 (71.9%) SNPs were selected and used for the analysis. The measures of the LD, square of correlation coefficient (r^2^) between SNP pairs, were calculated for each allele and the effective population size was determined based on r^2^ measures. The polymorphism information contents (PIC) and expected heterozygosity (HE) were 0.27 and 0.34, respectively. In LD, the most rapid decline was observed over the first 1 Mb. But r^2^ decreased more slowly with increasing distance and was constant after 2 Mb of distance and the decline was almost linear with log-transformed distance. The average r^2^ between adjacent SNP pairs ranged from 0.20 to 0.31 in each chromosome and whole average was 0.26, while the whole average r^2^ between all SNP pairs was 0.02. We observed an initial pattern of decreasing N_e_ and estimated values were closer to 41 at 1 ~ 5 generations ago. The effective population size (41 heads) estimated in this study seems to be large considering Jeju horse’s population size (about 2,000 heads), but it should be interpreted with caution because of the technical limitations of the methods and sample size.

## Background

According to the literature, horses began to be raised in Jeju Island before the Goryo Dynasty. However, historically in 1276 Mongolian Yuan Dynasty of China established a horse ranch in Jeju Island and 160 Mongolian horses were introduced to produce warhorse. Through adaptation to the harsh environment of the Jeju Island and long term isolation, Jeju horses have developed their own conformation. They have several coat colours and body size is smaller than that of Mongolian horse. Since 1960s due to the industrialization and the development of agricultural machines and means of transportation, demand for horses decreased. In 1986, dozens of Jeju horses with pedigree registry were designated as a natural monument (No.347) because of their historical importance. In May, 2000, Livestock Promotion Agency was designated as Jeju horse registration agency and Jeju horse registration started. Currently, about 2,000 heads of Jeju horses are being raised at local ranches. Domestic animals are well suited for genetic studies, since they enable comparisons of populations exposed to different selection criteria and environmental challenges [1, 2]. Jeju horses are very valuable animals to preserve historically and economically and it is very important to investigate unique genetic characteristics of Jeju horses [3, 4]. Jeju horses have been isolated for more than 700 years and it is estimated that their homozygocity of genotype increased by inbreeding and genetic drift. The increase of recessive homozygosity caused inbreeding and decreased growth and reproductive performance [5, 6]. Especially, average withers height of Jeju horse, approximately 122 cm, is shorter than that of Mongolian (140 cm).

As the rapid development of microarray technology, high density whole genome SNPs (SNP chip) became a strong tool for the researches of quantitative and population genetics. Recently, these genome-wide SNPs were commonly used for estimation of historical effective population size in livestock [7–13] and human [14, 15]. Closely-linked loci give information on population sizes over historical periods of time, while loosely-linked loci estimate population sizes in the immediate past [16–18]. Using high density SNPs, LD of many SNP pairs which have either close linkage or loose linkage by the distance between SNPs can be measured and used for estimation of historical effective population size.

This experiment was conducted to investigate the LD in population level and to estimate the effective population size for systemic preservation using genomic information of Juju horses.

## Material and methods

### Single-nucleotide polymorphism (SNP) data

**Figure 1:**
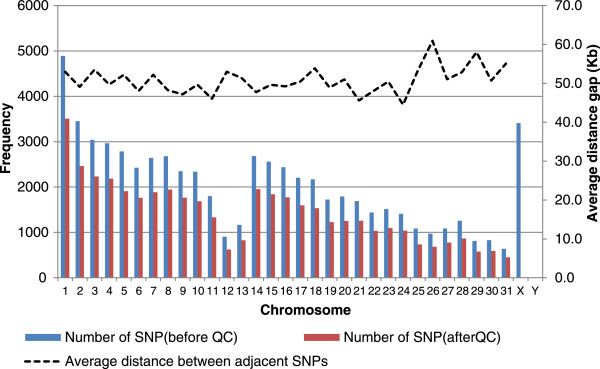
**Number of SNPs and average distance between adjacent SNPs per chromosome after quality control processes.**

DNA samples were obtained from 240 Jeju horses (racehorses) that were randomly chosen and had raced at the Jeju racing park and they were genotyped for the initial genome-wide scan using Equine SNP70 BeadChips (Geneseek, Lincoln, NE). Genomic DNA was isolated from nasal area according to the procedure of Performagene™-LIVESTOCK PG-AC1 Reagent Package (DNA Genotek INC, Canada). The quantity and quality of the genomic DNA was evaluated using 0.8% Agarose gel electrophosis and Nanodrop ND-100 electrophotometer. Genotyping was performed using the InfiniumHD iselect Custom BC Neogen_Equine_Community_Array (Illumina, USA), which contained 65,157 SNPs across the whole genome. Genomestudio softwareV.2011.1.9.4 (Illumina, USA) was used to call the genotypes from the samples. The chip includes 65,157 SNPs that are uniformly distributed on the 31 equine autosomes, X and Y chromosomes from the EquCab2 SNP database of the horse genome (Figure 1). We excluded the SNPs with a missing genotype rate of over 10%, minor allele frequency (MAF) of less than 0.05, and Hardy–Weinberg equilibrium (HWE) test P-value of less than 10^–6^ as a quality control procedure [13]. SNPs on the X and Y chromosomes and genotyped individuals with missing genotype rate over 10% were also excluded, remaining 44,426 autosomal SNPs from 218 heads for further analysis.

### Linkage disequilibrium (LD)

The measures of the LD were square of correlation coefficient (r^2^) between SNP pairs and calculated for each allele at locus A with each allele at locus B [7, 19].
1

Where D = P_AB_-P_A_P_B_ and P_A_, P_a_, P_B_ and P_b_ are the frequencies of alleles A, a, B and b, respectively.

### Effective population size

The effective population size was determined based on r^2^ measures. Because LD breaks down more rapidly over generations for loci further apart, LD at large distances reflects N_e_ at recent generations.
2

Where, N_e_ is effective population size and c is the recombination distance (in Morgans) between the SNPs. Equation (2) can be rearranged as follows [17, 20–22]:
3

Where, N_e_ is the effective population size t generations ago, c is the distance between markers in Morgans, r^2^_c_ is the mean value of r^2^ for markers c Morgans apart, and c = (2 t)^-1^. Megabase to centimorgan conversion rate was applied for generation grouping based on the result of Corbin et al. [21]. The estimation of LD measure and effective population size was used programs that we developed by GNU Fortran.

## Results and discussion

### Single-nucleotide polymorphism (SNP) data

Of the total 61,746 genotyped autosomal SNPs, 17,320 (28.1%) SNPs were excluded after quality control processes (missing genotype rate of >10%, minor allele frequency of <0.05 and Hardy–Weinberg equilibrium test P-value of <10^–6^) and finally, 44,426 (71.9%) SNPs were selected and used for the analysis. The minor allele frequencies (MAF) in each chromosome followed a uniform distribution and averaged to be 0.24 and the average *χ*^2^ value (p-value) of Hardy-Weinberg disequilibrium (HWE) test, polymorphism information contents (PIC) and expected heterozygosity (HE) were 1.32 (0.25), 0.27 and 0.34, respectively. The number of SNPs per autosome ranged from 452 to 3,509 and average distance between adjacent SNPs was 50.4 kb (Table 1), and their relationships are shown in Figure 1. The frequency of adjacent SNP pairs which are aparted between 10 Mb (Mega base pairs = 1,000,000 bp) and 100 Mb was 27,289 (61.4%), and that of adjacent SNP pairs less than 10 Mb was 14,764 (24.9%).

**Table 1:** **Simple statistics for single-nucleotide polymorphism (SNP) data by chromosome**

Chromosome	No. of SNPs	Mean				No. of SNP pairs	Mean
		Distance ^1^	MAF ^2^	HE ^3^	r-square ^4^		r-square ^5^
1	3,509	53.0	0.24	0.33	0.25	6,154,786	0.02
2	2,463	49.1	0.25	0.34	0.29	3,031,953	0.02
3	2,234	53.5	0.25	0.34	0.27	2,494,261	0.02
4	2,185	49.7	0.24	0.33	0.28	2,386,020	0.02
5	1,909	52.2	0.25	0.34	0.26	1,821,186	0.02
6	1,762	48.1	0.24	0.33	0.25	1,551,441	0.02
7	1,887	52.2	0.24	0.33	0.28	1,779,441	0.02
8	1,946	48.2	0.25	0.34	0.27	1,892,485	0.03
9	1,767	47.2	0.26	0.34	0.29	1,560,261	0.03
10	1,688	49.7	0.24	0.33	0.27	1,423,828	0.03
11	1,332	46.0	0.24	0.34	0.27	886,446	0.03
12	622	53.0	0.25	0.34	0.21	193,131	0.03
13	826	51.4	0.24	0.33	0.20	340,725	0.03
14	1,954	47.7	0.24	0.34	0.28	1,908,081	0.03
15	1,841	49.6	0.24	0.33	0.26	1,693,720	0.02
16	1,775	49.2	0.24	0.33	0.25	1,574,425	0.02
17	1,598	50.5	0.25	0.33	0.31	1,276,003	0.03
18	1,532	53.8	0.24	0.33	0.27	1,172,746	0.02
19	1,225	48.9	0.24	0.33	0.26	749,700	0.03
20	1,252	51.0	0.25	0.34	0.26	783,126	0.02
21	1,257	45.6	0.24	0.33	0.25	789,396	0.03
22	1,036	48.1	0.24	0.34	0.24	536,130	0.02
23	1,097	50.4	0.25	0.34	0.28	601,156	0.03
24	1,039	44.5	0.25	0.34	0.25	539,241	0.03
25	737	53.2	0.24	0.33	0.25	271,216	0.03
26	684	61.0	0.24	0.33	0.22	233,586	0.03
27	778	51.1	0.25	0.34	0.25	302,253	0.03
28	867	52.9	0.24	0.33	0.27	375,411	0.03
29	579	58.0	0.24	0.33	0.23	167,331	0.03
30	593	50.7	0.24	0.33	0.23	175,528	0.03
31	452	55.1	0.25	0.33	0.20	101,926.	0.03
Overall	44,426	50.4	0.24	0.34	0.26	38,766,939	0.02

^1^Kilo base pairs (Kb) between adjacent SNPs, ^2^minor allele frequency, ^3^Expected heterozygosity, ^4^between adjacent SNP pairs, ^5^between all SNP pairs.

### Linkage disequilibrium (LD)

**Figure 2:**
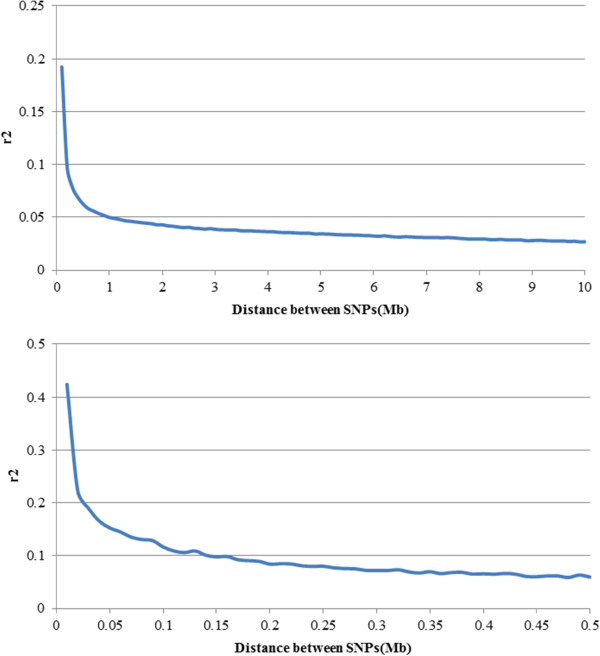
**Trend on r** ^**2**^ **between SNP pairs according to distance with all chromosomes.** (Upper) Distance range from 0 to 10 Mb. r^2^ values averaged using bins of 0.1 Mb. (Lower) Distance range from 0 to 0.5 Mb. r^2^ values averaged using bins of 0.01 Mb.

The results of this study provide an overview of LD in the Jeju Horse using a high density SNP panel. Linkage disequilibrium decreased with increasing distance between SNP pairs (Figure 2) and the most rapid decline was observed over the first 1 Mb. But r^2^ decreased more slowly with increasing distance and was constant after 2 Mb of distance and the decline in LD was almost linear with log-transformed distance [21]. The average r^2^ between adjacent SNP pairs ranged from 0.20 to 0.31 in each chromosome and whole average was 0.26, while the whole average r^2^ between all SNP pairs was 0.02 (Table 1).

According to reports [21, 23] in a sample of 817 and 24 Thoroughbred horses, LD in r^2^ decreased from 0.6 to 0.2 when the distance between markers increased to 0.5 Mb. The pattern of decline of LD with distance in our population was similar (Figure 2), but the LD observed was lower (0.49 ~ 0.07) when compared with other reports [21, 23].

Validation work by Corbin et al. [21] on their Thoroughbred (817 head) data suggests that our sample size of 218 heads is more accurate to obtain an unbiased result of LD in our population. On the other hand, the pattern and magnitude of decline of LD with distance at less than 10 Mb were almost similar and linkage disequilibrium declined more slowly in Jeju horse population than in Thoroughbred populations [21].

### Effective population size

**Figure 3:**
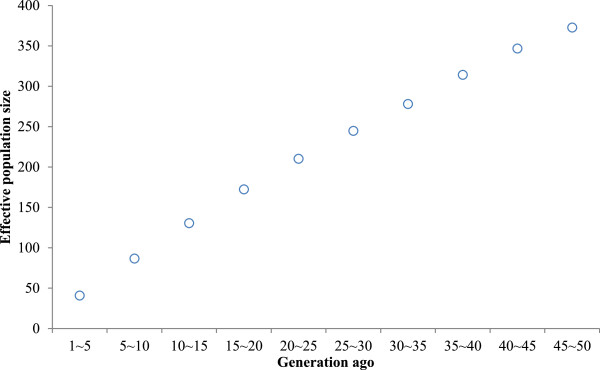
**Effective population size (N**_**e**_ **) plotted against generations in the past, truncated at 50 generations.**

We observed an initial pattern of decreasing N_e_ and estimated values were closer to 41 at 1 ~ 5 generations ago (Figure 3). This result is in agreement with the previous approach [17] by calculating historical N_e_, assuming linear population growth. The observed pattern showed a decrease in N_e_ upto around 1 ~ 5 generations. Corbin et al. [21] reported the effective population size (N_e_) was estimated to be 100 heads at 20 generations in Thoroughbreds and Cunningham et al. [24] calculated the effective number of studbook founders of the Thoroughbred to be 28.2 from pedigree analyses.

The 41 heads (N_e_) estimated in this study seems to be large considering Jeju horse’s population size. Currently, there is about 2,000 Jeju horses in Jeju Island and it may be difficult to interpret inflated N_e_. There may be a few speculations, such as an immigration event, a hybridization event or any combination of these. Therefore, it is useful to consider our observation in the context of what is known about the demographic history of Jeju horses. In 1986, 150 Jeju horses with pedigree registry were designated as a natural monument (No.347). In October, 1990, Jeju horse racing park was open and Jeju horse racing started and the names of various horses raised in Jeju was unified to Jeju horse. As the sales of Jeju horse racing park increased, the demand for Jeju horse increased and since the horses raised at ranches were selected as basic registered horses and included to Jeju horse management system, bloods of other breeds might be introduced.

On the other hand, since intensive selection for racing performance of Throughbred has been conducted for long period, the effective population size of Throughbred can be relatively small. However, for Jeju horse, fundamental effective population size can be larger than that of Throughbred since almost no selection has been conducted for Jeju horses. The effective population size (41 heads) estimated at 1 ~ 5 generations should be interpreted with caution because of the technical limitations of the methods and sample size.

## Conclusions

Jeju horses are very valuable animals to preserve historically and economically and it is very important to investigate unique genetic characteristics of Jeju horses for the stable maintenance. Also, we should make efforts to prevent inbreeding coefficient increase and to increase effective population size through the reduction of generation interval.
